# Wilms’ tumor suppressor gene (*WT1*) suppresses apoptosis by transcriptionally downregulating *BAX* expression in immature rat granulosa cells

**DOI:** 10.1186/s13048-014-0118-y

**Published:** 2014-12-10

**Authors:** Minji Park, Yuri Choi, Hyeonhae Choi, Jaesook Roh

**Affiliations:** Laboratory of Reproductive Endocrinology, Department of Anatomy and Cell Biology, College of Medicine, Hanyang University, San17 Haengdang-dong, Seongdong-gu, Seoul 133-791 South Korea

**Keywords:** *WT1*, Apoptosis, *Bax*, *bcl-2*, *Caspase*, Granulosa cell, Rat

## Abstract

**Background:**

The important role of *WT1* in early folliculogenesis was evident from its restricted expression pattern in immature follicles and from its involvement in transcriptional control of *inhibin-α* and FSH receptor. There is also considerable evidence that *WT1* is a potent inhibitor of apoptotic cell death in the developing kidney and male germ cells, suggesting that it could play a role in the regulation of follicle survival. Therefore, we evaluated if *WT1* involves in cell survival of granulosa cells (GCs) during the FSH-independent stage.

**Methods:**

GCs were obtained from small preantral follicles of immature rat ovary. *Bax* and *bcl-2* mRNA and protein levels in GCs transfected with *WT1* (−KTS) or *WT1* (+KTS) were analyzed by Real-time RT-PCR and immune-blotting analysis. Cell viability was measured with MTT assays and apoptosis was analyzed with *caspase* 3/7 activity and TUNEL assay. The mechanism by which *WT1* regulates *Bax* expression was investigated using Bax promoter-luciferase reporter assay and ChIP assays from GCs.

**Results:**

Here, we showed that *WT1* (−KTS) suppressed endogenous *Bax* transcript and protein expression, and this inhibition resulted from direct binding of *WT1* in the *Bax* promoter region in vivo. In addition, anti-apoptotic effects of *WT1* (−KTS) were demonstrated based on MTT assays, a sensitive bioluminescence *caspase* 3/7 assay and TUNEL assays. On the other hand, *WT1* has no role on *bcl-2* expression in GCs.

**Conclusion:**

These findings suggest that activation of *WT1* is necessary for maintenance of GC survival during early stage of follicles and *WT1* can play a role in protecting apoptosis through the regulation of upstream activator (*Bax*), as well as through regulation of downstream effecter (*caspase*s 3 and 7).

## Introduction

The fate of the follicles depends on the balance between anti- and pro-apoptotic factors that determine the rate of granulosa cell (GC) apoptosis. Although apoptosis can occur at any stage of follicular development, a growing follicle is more prone to apoptosis than arrested or early-stage follicles, and the majority of follicles become atretic during the antral stage [1]. The factors regulating follicular growth and survival appear to differ for small follicles and the more differentiated antral follicles. Further study of how the apoptotic genes are regulated may answer the questions of why quiescent follicles are not apoptotic, and why early-stage follicles are relatively resistant to apoptosis until they receive appropriate signals to initiate further growth.

The important role of *WT1* in early folliculogenesis is evident from the fact that its expression is restricted to immature follicles [2] and from its involvement in the transcriptional control of ovarian marker genes that encode *inhibin-α* [3] and the follicle stimulating hormone (FSH) receptor [4]. There is also considerable evidence that *WT1* is a potent inhibitor of apoptotic cell death in the developing kidney [5] and male germ cells [6], suggesting that it could play a role in the regulation of follicle survival. However, there is no direct evidence of an anti- or pro-apoptotic function of *WT1* protein, and its precise mechanism of action in the ovary is not well understood.

We hypothesized that *WT1* was required to regulate the transcription of the genes that provide a cell survival advantage to the GCs of the early preantral follicles in the FSH-independent stages of development. Here we investigated whether the expression of *WT1* was associated with changes in the expression of two apoptosis related genes, *bcl-2* and *Bax*, which function as the final common mediators of life and death [7]. In addition, we examined the effects of *WT1* on GC viability and apoptosis. A better understanding of the cellular signals that induce or prevent apoptosis may help us control follicular development and rescue more oocytes from quiescent early follicles.

## Materials and methods

### Animals

Immature female Sprague–Dawley rats were obtained from Samtako Biokorea (Kyunggi, South Korea). All animals were housed under controlled humidity, temperature, and light conditions, and fed standard rat chow *ad libitum*. Animal care was consistent with institutional guidelines, and the Hanyang University ACUC committee approved all procedures involving animals (HY-IACUC-09-034).

### Preparation and culture of granulosa cells

GCs were obtained by puncture of the ovaries of immature 25-day-old rats (body weight, 55–65 g) previously primed with diethylstilbestrol. The ovaries were punctured in Leibovitz L-15 medium. Ovarian debris was removed, and the remaining medium containing GCs was collected after low-speed centrifugation at 500 × g for 10 min. The GCs were dispersed by repeated washing and suspended in growth medium (McCoy’s 5a supplemented with 2 mM L-glutamine, 100 U/mL penicillin, and 100 μg/mL streptomycin). McCoy’s 5a medium (modified) and Leibovitz L-15 medium were obtained from GIBCO (Santa Clara, CA, USA). Penicillin and streptomycin were obtained from Sigma (St. Louis, MO, USA). Recombinant human FSH (Org 32489E) was from NV Organon (Oss, The Netherlands).

### Plasmid construction and transfection

Full-length mouse *WT1* cDNA (−KTS) or (+KTS) was subcloned into the pCMV5 expression vector under the control of the CMV promoter and the GH-pA signal, [*WT1* (−KTS)] or [*WT1* (+KTS)]. Granulosa cells (5 × 10^5^ viable cells/well) were grown in culture medium supplemented with 10% fetal bovine serum (FBS) for 2 h. The medium was then changed to serum-free medium and the cells were transfected with expression and/or reporter plasmids using Lipofectamine 2000 reagent (Invitrogen, Carlsbad, CA, USA) according to the manufacturer’s instructions. The cells were used for the experiment at sixteen hours after transfection. At the end of growth they were frozen for RNA or protein extraction.

To evaluate promoter activity induced by *WT1* overexpression, cells were cotransfected with the *Bax* (−2673 to +1 bp of the 5’ flanking sequence of the mouse *Bax* gene) promoter-luciferase reporter plasmid *Bax*-Luc (250 ng) with *WT1* (−KTS), or *WT1* (+KTS) cDNA (100 ng) or empty vector as a balancer. The p-Rous sarcoma virus (RSV)-β-galactosidase (gal) vector (50 ng) containing the lacZ gene encoding β-gal driven by the RSV long terminal repeat was used as an internal control to correct for differences in transfection efficiency. Sixteen hours after transfection, the cells were incubated in FSH (50 ng/mL) or control medium for 16–24 h, then harvested, lysed, and assayed for luciferase activity.

To harvest the cells, lysis buffer (200 μL) (Promega) was added to each well and 30 μL of supernatant was used to detect luciferase activity on a Monolight 2010 luminometer (Analytical Luminescence Laboratory, San Diego, CA, USA). 50 μL of cell lysate was also used to measure β-gal activity. The activity of the promoter is expressed as the ratio of relative light units /β-gal activity.

### Real time RT-PCR

Total RNA was isolated with an RNeasy extraction kit (Qiagen Inc., Valencia, CA, USA). 1 μg aliquots of total RNA were annealed (5 min at 70°C) to oligo(dT)_18_ primers and reverse transcribed using an Advantage RT-for-PCR kit (BD Biosciences; Clontech, Palo Alto, CA, USA). The primers were designed for the mRNA sequences of *Bax* and *bcl-2* using the Primers Express program (PE Applied Biosystems, Foster City, CA, USA): *Bax* forward, 5’-TGTTTGCTGATGGCAACTTC-3’ and reverse, 5’-GATCAGCTCGGGCACTTTAG-3’ (GenBank accession no. NM_017059.1); *bcl-2* forward, 5’-GGGATGCCTTTGTGGAACTA-3’ and reverse, 5’-CTCACTTGTGGCCCAGGTAT-3’ (GeneBank accession no. NM_016993.1). *GAPDH* was amplified using 5’-GCTGGCATTGCTCTCAATGACA-3’ (forward) and 5’-TCCACCACCCTGTTGCTGTA-3’ (reverse) (GenBank accession no. NM_017008) to normalize each reaction (amplification product sizes 104, 138, and 83 bps for *Bax*, *bcl-2*, and *GAPDH*, respectively). Real-time polymerase chain reactions were carried out in 25 μL volumes using SYBR Green Supermix (BioRad, Hercules, CA, USA) in an iCycler Thermal Cycler (BioRad). Samples were run in triplicate in 96-well optical plates (BioRad) and mean values were compared with the control values (empty vector-transfected cells) to calculate the relative amount of transcript.

### Western blotting

**Figure 1:**
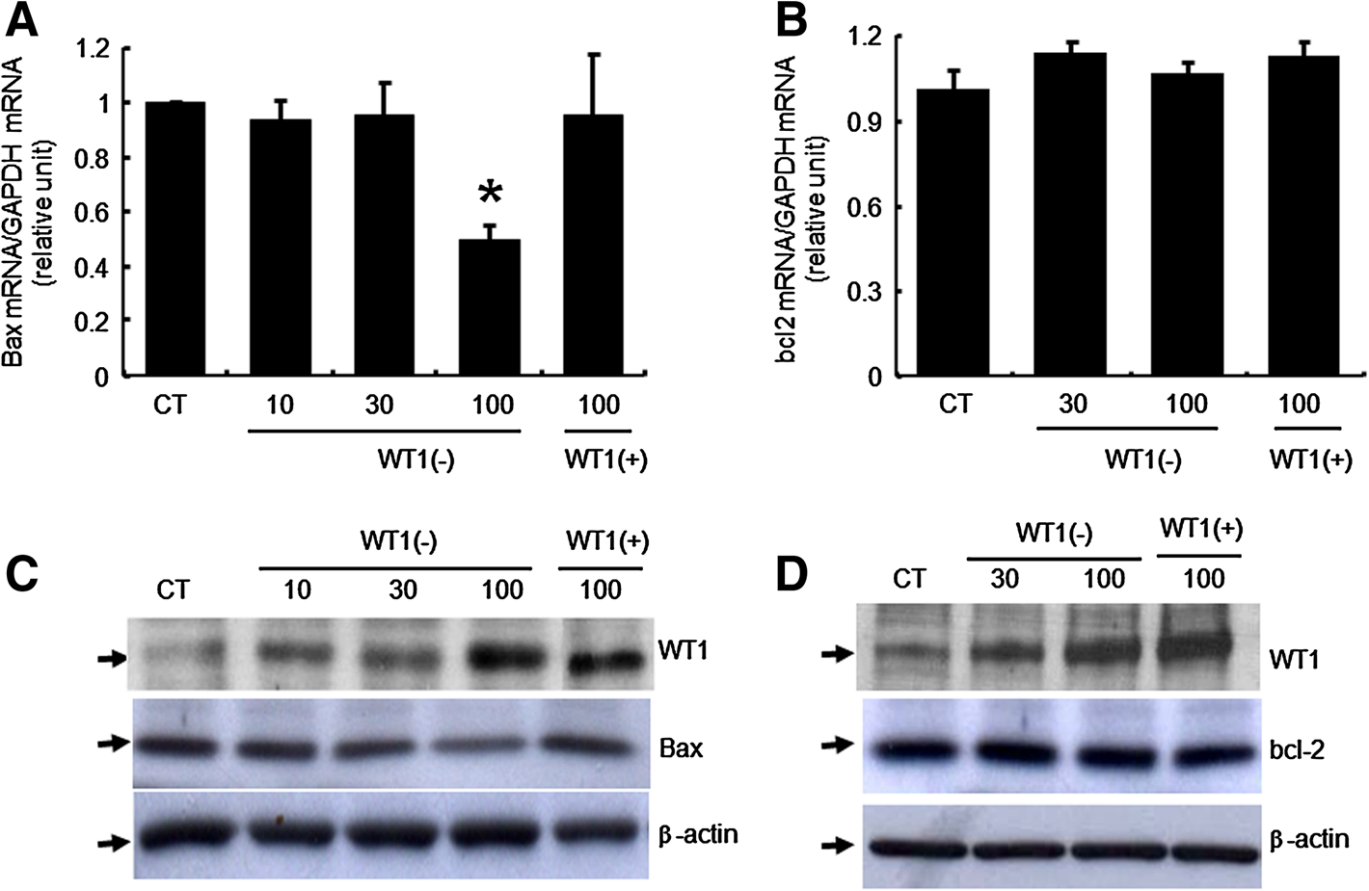
**Regulation of** ***Bax*** **and** ***bcl-2*** **expression by** ***WT1*** **in immature rat GCs.** Real-time RT-PCR analysis of **(A)** *Bax* and **(B)** *bcl-2* mRNA levels in GCs transfected with increasing amounts of *WT1* (−KTS) (10, 30, 100 ng/well) or *WT1*(+KTS) (100 ng/well). *GAPDH* was used to normalize each reaction. Values are calculated as fold changes relative to control (CT), and are expressed as the mean ± SD of three independent experiments, each performed in triplicate. CT, cells transfected with empty vector. *p < 0.05 compared to control. Western blot analysis of **(C)** *Bax* and **(D)** *bcl-2* protein levels. Lysates were immunoblotted with anti-*WT1* antibody (C-19; Santa Cruz Biotechnology, Inc.), anti-*Bax* antibody (B-9, SC 7480), or anti-*bcl-2* antibody (C-2, SC 7382). Arrows indicate bands corresponding to *WT1* (52 kDa), *Bax* (22 kDa), *bcl-2* (26 kDa) and β-*actin* (42 kDa), respectively.

To investigate *Bax* and *bcl-2* protein regulation by *WT1*, GCs transfected with *WT1*(−KTS) or *WT1*(+KTS) were harvested after 24 h culture and washed once on ice with cold PBS before lysis in Laemmli buffer containing β-mercaptoethanol. The cells were gently sonicated on ice for 15 sec with an MSE sonicator (Sanyo Corp., Osaka, Japan) and boiled for 3 min. Proteins were separated on 8% SDS-PAGE gels and electroblotted onto Hybond-P membranes (Amersham Pharmacia Biotech, Arlington Heights, IL, USA). To detect *WT1*, *Bax*, and *bcl-2*, the membranes were blocked for 1 h at room temperature in TBS-0.1% Tween containing 5% fat-free dry milk, and incubated with anti-*WT1* antibody (C19; Santa Cruz Biotechnology, Inc., Santa Cruz, CA, USA), anti-*Bax* antibody (B-9, SC 7480), or anti-*bcl-2* antibody (C-2, SC 7382) diluted 1:500 for 3 h at room temperature. The membranes were washed and blotted with peroxidase-conjugated donkey anti-rabbit secondary antibody (1:8,000) (Boehringer Mannheim, Indianapolis, IN, USA) for *WT1*, or with peroxidase-conjugated goat anti-mouse secondary antibody (1:5,000) (W402B, Promega, Madison, WI, USA) for *Bax* and *bcl-2*. Immunolabeled proteins were detected using an enhanced chemiluminescence kit (Amersham Pharmacia Biotech). The bands corresponding to *WT1*, *Bax*, and *bcl-2* proteins (respectively, 52, 22, and 26 kDa) are indicated in Figure 1C and D. To ensure the cell lysates were loaded equally, the blots were stripped and incubated with β-*actin* (1:1,000 dilution; Sigma, St. Louis, MO, USA). Data were collected from at least two independent experiments.

### Analyses of cell viability and apoptosis

Cell viability was determined using an MTT [3-(4,5-dimethythiazol-2-yl)-2,5-diphenyltetrazolium bromide] assay kit (Cayman Chemical Company, Ann Arbor, MI, USA). The conversion of MTT into the aqueous soluble formazan product is accomplished by dehydrogenase enzymes found in metabolically active cells. GCs were cultured in McCoy 5a serum-free medium with or without FSH or 10% FBS for 24 h after transfection. After addition of tetrazolium salt solution and incubation at 37°C for 2 h, the quantity of the formazan product was measured by the absorbance at 570 nm. The experiment was repeated three times, and the data were expressed as fold changes relative to the control values measured with empty vector-transfected cells cultured for 24 h in the absence of serum.

Apoptosis was subsequently assessed with the *caspase* 3/7 activity assay using a luminescence assay kit (Caspase-Glo 3/7 assay; Promega). GCs were cultured in McCoy 5a serum-free medium with or without FSH or 10% FBS for 24 h after transfection. Then proluminescent Caspase-Glo 3/7 reagent was added to each well, and the cells were gently mixed and incubated at room temperature for 1 h. Luminescence was measured on a Monolight 2010 luminometer (Analytical Luminescence Laboratory, San Diego, CA, USA). As for the MTT assay, the data were expressed as fold changes relative to the control.

### Terminal deoxynucleotidyl transferase-mediated dUTP-biotin nick end labeling (TUNEL) assay

Apoptosis was analyzed by 3’-end labeling of DNA fragments in situ using the ApopTag In-Situ Apoptosis Detection kit (Oncor, Gaithersberg, MD) according to the manufacturer’s protocol. In addition, we performed fluorescent staining for *WT1* protein to confirm overexpression of *WT1* in transfected GCs. GCs (1 × 10^5^ cells) were plated onto 2 × 2 cm coverslips at 6-well plate and transfected with *WT1* or empty-vector for 6 h and then serum starved for another 36 ~ 48 h. After then, cells were fixed with 4% paraformaldehyde for 10 min at room temperature and washed twice in PBS. The cells were then incubated with terminal deoxynucleotidyl transferase and digoxigenin-11 dUTP in a humidified chamber at 37°C for 1 h or polyclonal anti-*WT1* antibody (1:100) for 2 h at room temperature. The cells were washed with PBS and incubated with antidigoxigenin-fluorescein antibody for 1 h for TUNEL or a Alexa Flour 594 goat antirabbit IgG (1:200)(Invitrogen, Carlsbad, CA, USA) for 30 min for *WT1*. The cells were then washed with buffer and nuclear DNA was counterstained with 4’,6-diamidino-2-phenylindole (DAPI)(Vector Laboratories, Inc., Burlingame, CA, USA). We counted the number of apoptotic cells using a Leica TCP SP5 confocal microscope (Leica, Heidelberg, Germany). We randomly selected 4 ~ 6 magnified fields (X200) and calculated the average number of apoptotic cells per field. Apoptosis was expressed as the percentage of TUNEL-positive nuclei per field. These experiments were repeated five times.

### Chromatin immunoprecipitation (ChIP) analysis

**Figure 2:**
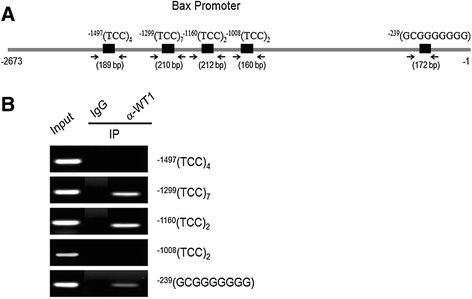
**Evidence of** ***WT1*** **binding in the** ***Bax*** **promoter region** ***in vivo*** **. (A)** Schematic showing the *Bax* promoter and the target region tested in ChIP assay. The locations of primers used to amplify DNA fragments spanning *WT1* transcription binding sites were designated in the Bax promoter region. **(B)** ChIP detection of *WT1* transcription factor binding to the *Bax* promoter region in immature GCs. ChIP assays were performed using DNA extracted from GCs obtained at 36–48 h after transfection. One tenth of the chromatin was kept as input DNA control (Input) before immunoprecipitation. Immunoprecipitations were performed with *WT1* antibody, or normal rabbit IgG served as a negative control. DNAs were analyzed by PCR using primers indicated previously. WT1^−1299^ (210 bp) and WT1^−1160^ (212 bp) DNA fragments containing *WT1* transcription factor binding sites were enriched in chromatin samples immunoprecipitated with *WT1* antibody as well as input DNA. A representative gel picture from at least four independent experiments is shown.

ChIP assay was performed on *WT1* in the *Bax* promoter region using a ChIP kit (Upstate Biotechnology, Inc., Lake Placid, NY) according to the manufacturer’s protocol with minor modifications. Briefly, immature rat GCs (2×10^7^) were transfected with *WT1*(−KTS) using lipofectamine 2000, cultured in McCoy 5a serum-free medium for 24 h, and cross-linked with 1% formaldehyde for 10 min at room temperature. The reaction was terminated by the addition of glycine (final concentration, 0.125 M) for 5 min at room temperature. Cells were pelleted by centrifugation and lysed in 1 ml ice-cold lysis buffer containing protease inhibitor cocktail (Roche Applied Science, Indianapolis, IN, USA). The lysates were sonicated on ice at a 250 W power level for 25 min with 15-sec sonication and 30-sec intervals with a Bioruptor KR (CosmoBio Co., Ltd., Tokyo, Japan) to obtain DNA fragments of an average length of approximately 100–500 bp. Chromatin was immunoprecipitated overnight at 4°C with anti-*WT1* antibody (10 ug/reactions; Santa Cruz Biotechnology, Inc., Santa Cruz, CA, USA) or normal rabbit IgG (5 ug/reaction; Santa Cruz Biotechnology, Inc., Santa Cruz, CA, USA) as a negative control. Immune complexes were collected using protein G-agarose slurry for 2 h at 4°C with rotation and sequentially washed for 3 min each in low salt wash buffer, high salt wash buffer, LiCl wash buffer, and two washes with Tris/EDTA buffer. Precipitates were then extracted two times with elution buffer. Eluates were pooled, and cross-linking was reversed by incubation at 65°C overnight. Unbound proteins were digested with proteinase K (Promega) for 2 h at 45°C, and chromatin was purified using the DNA Clean-up kit (GeneAll Biotechnology, Seoul, Korea). DNA was analyzed by PCR using the primers designed to amplify fragments of the *WT1* motif in the *Bax* promoter [see Figure 2A, WT1^−1497^(forward 5’-GGC CTG CTG CTA CTT CAC AT-3’, reverse 5’-TTT TAA TCC CAG CCC TCA GA-3’), WT1^−1299^(forward 5’-TAG TCC AGG CTG ACC TCG AA-3’, reverse 5’-ATG GTG GCC CAT ACC TGT AA-3’), WT1^−1160^(forward 5’-CAT CCT AGG CTG GCT TCA GA-3’, reverse 5’-CCT TCT CAC CTC ACC ATA CCT C-3’), WT1^−1008^(forward 5’-GAG GTA TGG TGA GGT GAG AAG G-3’, reverse 5’-TCT CTC TCC TCC TTT CCC AAA-3’), and WT1^−239^(forward 5’-ATA TCC CAG GCA AGC TTT GA-3’, reverse 5’-GCC GCG GGT ACT AAA TGA AC-3’)]. After 25- to 28-cycle amplification, PCR products were run on a 1.5% agarose gel, stained with ethidium bromide, and visualized under UV light.

### Data analysis

All results are expressed as the mean ± SD of duplicate measurements of triplicate cultures, and each experiment was repeated at least three times. Statistical significance was determined by Mann–Whitney *U*-test for two group comparison and Kruskal-Wallis one-way analysis of variance for multiple group comparisons. Significance was accepted at p < 0.05.

## Results

### Effects of *WT1* overexpression on basal *Bax* and *bcl-2* transcript and protein levels

To determine whether *WT1* regulates the endogenous expression of pro-apoptotic genes directly, we analyzed primary GCs expressing either *WT1* (+KTS) or *WT1* (−KTS). Since insertion or removal of the three amino acid sequence (KTS) changes the DNA-binding specificity of *WT1* [8,9], it was important to compare the effects of the two isoforms in GCs. Markers of apoptosis (i.e. *Bax* or *bcl-2*) were detected using real time RT-PCR and Western blotting. Overexpression of *WT1* (+KTS) and *WT1* (−KTS) in GCs was confirmed, and their corresponding immunoreactive bands (52 ~ 54 kDa) are shown in Figure 1C and D (upper lane). Total RNA was isolated from the cells expressing either the *WT1* (−KTS) or the *WT1* (+KTS) isoform. Interestingly, in the GCs expressing the *WT1* (−KTS) isoform, we observed significantly decreased levels of *Bax* transcripts, while there was no significant effect in the GCs expressing *WT1* (+KTS) (Figure 1A). It was important to determine whether this decrease led to a subsequent decline in the levels of *Bax* protein. To address this question, total proteins were isolated from GCs expressing either *WT1* (+KTS) or *WT1* (−KTS) proteins. Consistent with the findings of real time RT-PCR, the cells overexpressing *WT1* (−KTS) contained less *Bax* protein than the empty vector-transfected cells (Figure 1C), while the GCs expressing the *WT1* (+KTS) isoform had the control level of *Bax* (Figure 1C). The levels of *bcl-2* transcripts and protein were not altered by overexpression of either of the two *WT1* isoforms (Figure 1B and D).

### Effect of *WT1* overexpression on GC viability under serum deprivation

Despite its tumor suppressor function, *WT1* prevents programmed cell death in some cell types but promotes it in others [10–12]. Thus, we were interested to find out whether *WT1* protein altered sensitivity to apoptotic stimuli. To investigate the role of *WT1* in GC viability, we employed the MTT assay to monitor the activity of mitochondrial reductase [13]. GCs transfected with *WT1* were cultured with or without serum or FSH for 24 h. The purpose of removing the serum was to induce spontaneous onset of GC apoptosis because serum has shown to contain growth factors associated with a significant decrease in pro-apoptotic proteins [14]. On the other hand, FSH is the main physiological regulators of ovarian follicle survival and sufficient FSH concentrations are critical for survival of follicles [15,16].

**Figure 3:**
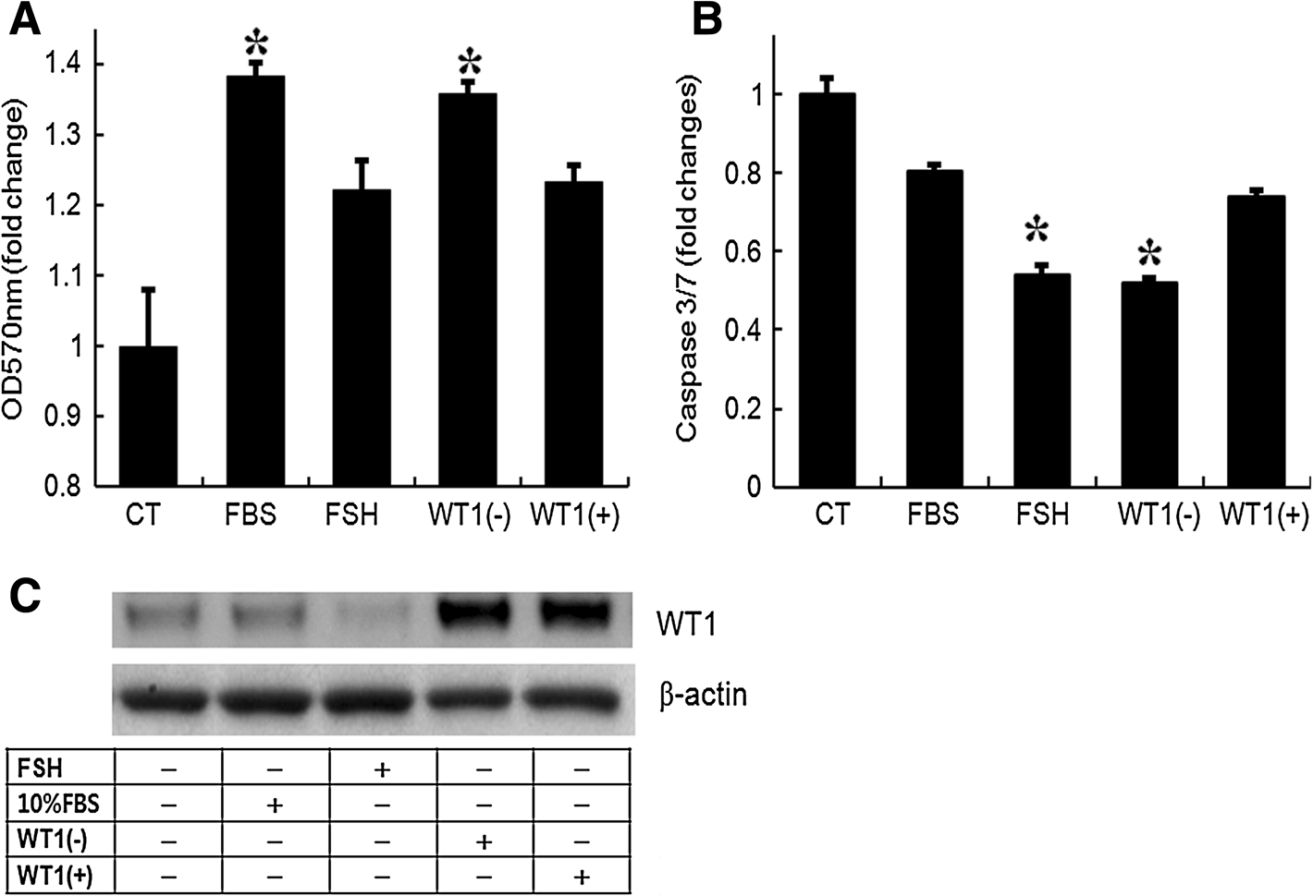
**Regulation of cell viability by overexpression of** ***WT1*** **(−KTS), but not** ***WT1*** **(+KTS). (A)** MTT assays of immature rat GCs transfected with one of the two *WT1* isoforms. GCs transfected with *WT1* or empty vector were incubated with or without serum for 24 h. Some cells were treated with FSH (50 ng/mL) as a positive control for survival factors. After the cells were incubated with tetrazolium salt solution for 2 h, the quantity of formazan product was determined from the absorbance at 570 nm. Each bar represents the fold change compared to control (CT). **(B)** analysis of *caspase* 3/7 activity. GCs transfected with *WT1* or empty vector were incubated with or without serum. Some cells were treated with FSH (50 ng/mL) as a positive control for apoptosis suppressors. 24 h later, *caspase* 3/7 activity was measured with the bioluminescence assay. **(C)** Immunoblot analysis of *WT1* protein in cultured GCs lysates to confirm *WT1* overexpression. Bands corresponding to *WT1* (52 kDa), and β-*actin* (42 kDa), respectively. CT, empty vector-transfected cells cultured without serum for 24 h. *WT1* (−KTS) (100 ng/well); *WT1* (+KTS) (100 ng/well); FBS, 10% fetal bovine serum. Data are expressed as the mean ± SD of three separate experiments. *p < 0.05 compared to control.

As shown in Figure 3A, the effect of *WT1* (−KTS) overexpression on MTT activity was significant (p = 0.029 compared to the control), and as potent as that of 10% FBS. In addition, we confirmed the role of FSH and serum as survival factors in immature GCs; addition of serum had a more potent effect than FSH, which may be related to the lower numbers of FSH receptors in immature GCs. Overexpression of *WT1* (+KTS) also increased MTT activity, but the effect was not statistically significant (p > 0.05).

### Effects of *WT1* overexpression on apoptosis in cultured GCs

To see whether the enhanced cell viability was due to suppression of apoptosis, we analyzed apoptosis quantitatively by measuring *caspase* 3/7 activity. After incubation of the transfected cells for approximately 16–24 h, GCs were cultured with or without serum or FSH. The enzymatic activities measured in each group were compared with the activities in the cells cultured in serum-free conditions (control). As shown in Figure 3B, in the GCs cultured with 10% FBS, *caspase* 3/7 activity was 0.8-fold of the activity in cells cultured in serum-free conditions (control) (p > 0.05). Interestingly, overexpression of *WT1* (−KTS) caused an approximately 0.5-fold decrease in *caspase*-3/7 activity compared to the cells cultured in serum-free conditions (p = 0.0026), indicating that *WT1* (−KTS) had an inhibitory effect on GC apoptosis induced by serum deprivation. Moreover, this inhibitory effect was as potent as that of FSH. *WT1* (+KTS) overexpression also decreased *caspase* 3/7 activity as much as 10% FBS, although the resulting activity was not significantly different from the control (p > 0.05). In summary, the expression of *WT1* (−KTS) in GCs seems to confer significant resistance to apoptotic cell death induced by serum deprivation, an effect as potent as FSH treatment.

**Figure 4:**
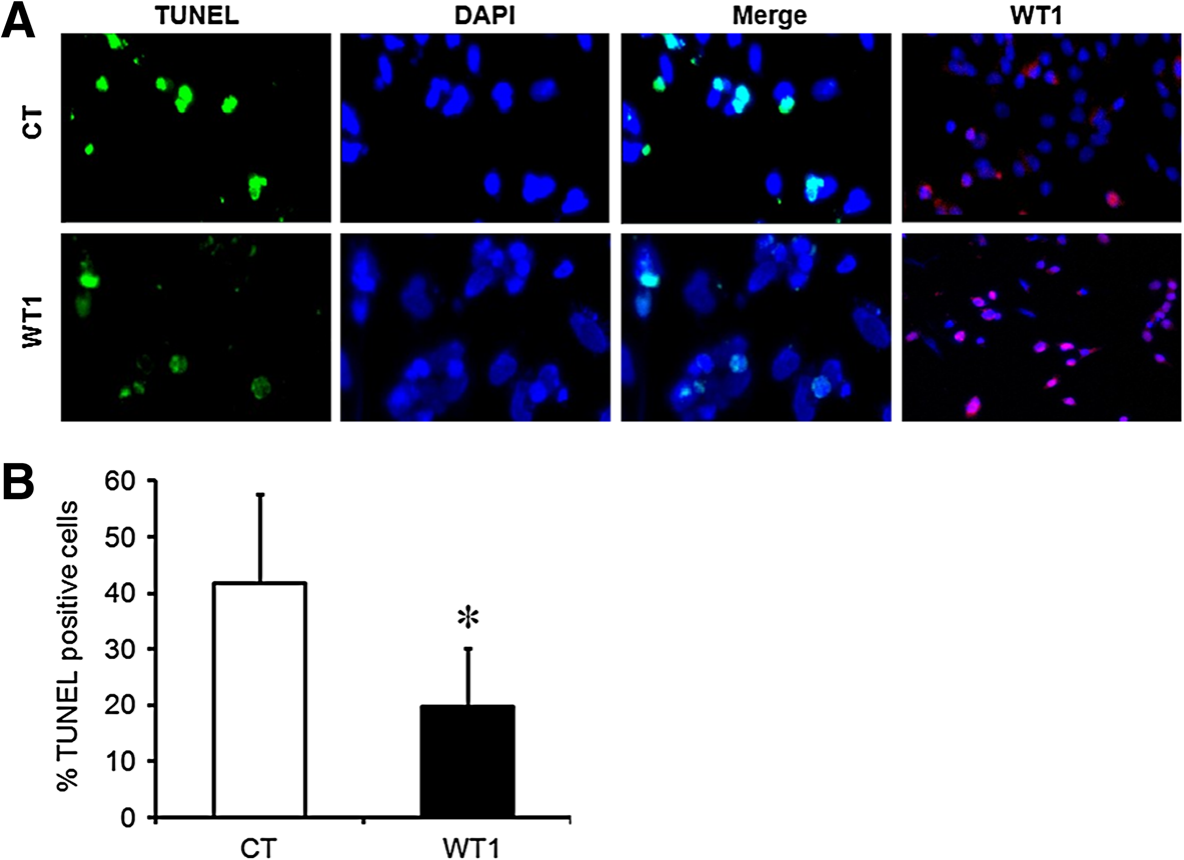
***WT1*** **suppresses granulosa cell apoptosis. (A)** GCs transfected with *WT1*(−KTS) or empty vector were stained with TUNEL or *WT1* antibody to visualize apoptotic cells (green) or *WT1* protein (pink: merge with DAPI) and counterstained with DAPI to confirm nuclear status (blue). Representative fluorescence confocal microscopy images are shown. Magnification; X100 or X200. **(B)** Frequency of TUNEL-positive nuclei in GCs transfected with empty vector or *WT1*(−KTS). Data represent mean ± SD of five independent experiments. CT, empty vector-transfected cells; WT1, GCs transfected with *WT1* (−KTS)(100 ng/well). * p < 0.05 vs. EV.

In addition, the effects of *WT1*(−KTS) on the incidence of GC apoptosis were determined by TUNEL staining in GCs (Figure 4). This was carried out in transfected GCs induced to undergo apoptosis in serum-free cultures [14]. *WT1* protein expression was profoundly increased in *WT1* transfected GCs and confirmed efficient transfection (Figure 4A). The percentage of apoptotic cells significantly decreased at 36–48 h of culture in *WT1* transfected cells as compared with the control (CT vs. WT1; 41.9 ± 15.7% vs. 19.7 ± 10.3%)(P < 0.01)(Figure 4B). These results showed that apoptosis was suppressed in GCs transfected with *WT1* compared with the control, suggesting that *WT1* is involved in GCs survival by regulating apoptosis.

### Role of *WT1* (−KTS) in suppressing *Bax* promoter activity

**Figure 5:**
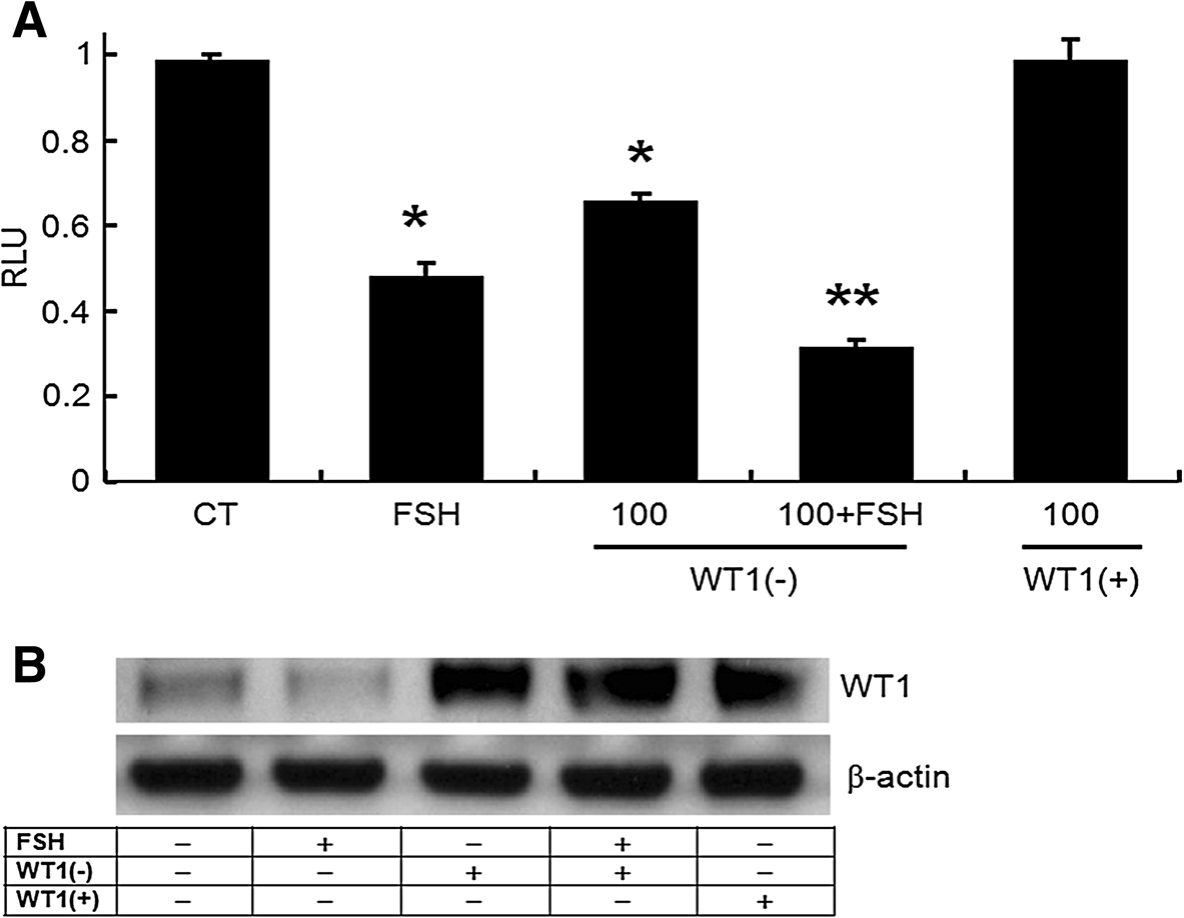
***WT1*** **(−KTS) suppression of** ***Bax*** **promoter activity in immature rat GCs. (A)** Cells were co-transfected with the *Bax* promoter luciferase reporter construct (250 ng/well) and either *WT1* (+KTS) or (−KTS) (100 ng/well), and cultured for 24 h. FSH (50 ng/mL) was used as a positive control for *Bax* repressor. Cell lysates were assayed for the activity of the luciferase reporter gene. Luciferase activity is expressed as relative light units (RLU) and normalized based on β-gal activity in co-transfected cells. Values are calculated as fold-changes relative to control (CT), and are expressed as the mean ± SD of three independent experiments, each performed in triplicate. **(B)** Immunoblot analysis of *WT1* protein in cultured GCs lysates to confirm *WT1* overexpression. Bands corresponding to *WT1* (52 kDa), and β-*actin* (42 kDa), respectively. CT, control (cells transfected with *Bax* promoter only). *p < 0.05 compared to control; **p < 0.05 compared to FSH.

The presence of potential binding elements for *WT1* in the *Bax* promoter suggested that *WT1* might affect *Bax* expression in the ovary (Figure 2A). Transient transfection assays were performed to determine whether the decrease in *Bax* levels, observed when *WT1* (−KTS) was overexpressed, was due to the ability of *WT1* (−KTS) to reduce *Bax* promoter activity. For comparing luciferase activities, cells transfected only with the *Bax* promoter construct, without any treatment, constituted the control group. Increased level of *WT1* protein expression in *WT1* transfected GCs was confirmed (Figure 5B). As shown in Figure 5A, the *WT1* (−KTS) transfected cells displayed a significantly reduced level of *Bax* promoter activity (0.6-fold of the control group) (p < 0.05). As expected, FSH significantly suppressed *Bax* promoter activity (0.5-fold of the control) and the addition of FSH to *WT1* (−KTS) transfected cells further inhibited *Bax* promoter activity (0.3-fold of the control) (p < 0.01). Conversely, *WT1* (+KTS) failed to affect *Bax* promoter activity. Collectively, these results provide evidence that the *Bax* promoter is transcriptionally down-regulated by the *WT1* (−KTS) isoform but not by the *WT1* (+KTS) isoform.

### Identification of the *WT1*(−KTS) binding sites on the *Bax* promoter region in GCs

To determine whether *WT1* directly regulates *Bax* expression, we first analyzed the putative promoter region [~2.7-kb upstream of transcription start site] of the *Bax* gene using a web-based transcription factor prediction program. This analysis revealed the presence of several putative *WT1* binding sites, with five consensus binding sites (Figure 2A). To examine whether *WT1* specifically binds to these candidate sites in the *Bax* promoter in vivo, ChIP assays were performed using immature GCs. PCR fragments [210 bp (−1394/-1185), 212 bp (−1253/-1042) and 172 bp (−331/-160)] containing the *WT1* binding sequence [WT1^−1299^(TCC)_7_, WT1^−1160^(TCC)_2_ and WT1^−239^(GCGGGGGGG), respectively] in the proximal promoter region were enriched in chromatin samples treated with *WT1* antibody compared with normal rabbit IgG, demonstrating the in vivo binding of *WT1* on the TCC repeats of the middle region of *Bax* promoter [^−1160^(TCC)_2_, ^−1299^(TCC)_7_] and the GNGGGGGGG in the proximal (Figure 2B), whereas no PCR fragment was amplified in the proximal and distal (TCC)_n_ region [^−1008^(TCC)_2_, ^−1497^(TCC)_4_].

## Discussion

The apoptotic death of GCs underlies the initiation and progression of follicular atresia [17]. A number of factors are thought to regulate the apoptosis of ovarian GCs [18], but relatively little is known about the survival of preantral follicles compared with the later stages of development. Although responsiveness to FSH plays a critical role in the follicular transition and follicle survival in the ovary [15,16], it is insufficient to prevent apoptosis during the initial, FSH-independent stages. In fact, the early antral follicles are more susceptible to apoptosis than preantral follicles [1,19]. Thus, our main aim was to identify how early-stage follicles remain healthy and non-apoptotic. To this end, we investigated the effects of *WT1* on the expression of pro-apoptotic genes and on cell survival in immature GCs.

It is widely accepted that the relative levels of tumor suppressors, apoptotic proteins, and survival factors in GCs determine the fates of early-stage follicles [20]. *WT1*, a member of the zinc-finger family of transcription factors, is highly expressed in GCs in early-stage follicles. Its expression undergoes dynamic changes during folliculogenesis, alongside changes in the spatial and temporal expression of the FSH receptor gene [3]. Once the early antral follicles begin expressing FSH receptors, they become dependent on FSH stimulation for survival [1], and *WT1* expression declines [2]. This stage-dependent regulation of *WT1* and its target genes, including the FSH receptor [4], suggests that a functional link between *WT1* and FSH signaling promotes the transition of follicles from small to more differentiated. The role of FSH as the main physiological regulator of ovarian follicle cell proliferation and survival is well established [18]. Since FSH acts as a key survival factor for the growing follicles [1], the small increase in cell numbers observed after FSH treatment (Figure 3) may be the result of a low responsiveness to FSH of immature GCs obtained from small preantral follicles. As a follicle grows, expression of the FSH receptor continues to increase [21] and FSH can exert its effects on downstream molecules, including *WT1* [22]. Therefore, given that *WT1* enhanced GC survival in this study, it is reasonable to assume that *WT1* plays a role in preventing apoptosis during the FSH-independent stages, until FSH takes on that role.

*Bcl-2* family proteins, including *bcl-2* and *Bax*, regulate the release of pro-apoptotic factors, and in particular of cytochrome c, from the mitochondrion into the cytosol [23], which is an almost universal event during apoptotic cell death. The product of the *bcl-2* gene prevents apoptosis, and *Bax* opposes *bcl-2* action [24]. The relationship between *WT1* and the *Bcl-2* family of proteins during GC apoptosis has not been well studied. Previously, *WT1* activation was shown to regulate several pro-apoptotic genes, including *Bax*, in non-gonadal cells [10,12,25]. *Bax* expression was shown to be regulated differently depending on cell lineage and isoforms. For instance, *Bax* expression was significantly decreased by either of the two *WT1* isoforms in breast cancer cells [25], whereas it was not changed in leukemic cells [11]. In the cells obtained from the ovary or the uterus, the *WT1* (−KTS) isoform was reported to be more potent than *WT1* (+KTS) as a regulator of ovarian marker genes [3,4]. Likewise, our results demonstrated a suppressive role of *WT1* (−KTS), but not of *WT1* (+KTS), on *Bax* expression in GCs. A possible explanation is that the KTS insertion disrupts the critical spacing between the zinc fingers resulting in a severe reduction of binding to the consensus *WT1* binding site [26]. However, a direct interaction between *WT1* and the *Bax* promoter has not been previously demonstrated. The *WT1* (−KTS) isoform binds to GC-rich sites (known as EGR1, WRE, and WTE) and to (TCC)n repeat elements [27]. An analysis of genomic sequences demonstrated the presence of potential binding elements for *WT1* (−KTS) in the *Bax* promoter region, as shown in Figure 2A. We also showed that *Bax* inhibition coincided with increasing levels of *WT1*, and FSH in cells overexpressed *WT1* (−KTS) further inhibited *Bax* promoter activity in GCs, as shown in Figure 5A. In addition, our results demonstrated that *WT1* (−KTS) down-regulates endogenous *Bax* levels by transcriptionally regulating the *Bax* promoter through a high-affinity *WT1*-binding site (Figure 2B).

On the other hand, *WT1* appears to have no role in *bcl-2* expression in GCs, even though it was shown to either suppress or activate *bcl-2* in non-gonadal cells [10,28]. Apoptosis in follicles in vitro is mainly associated with a marked increase in *Bax* levels with no significant change in *bcl-2* [29]. Since the levels of *bcl-2* stay relatively constant, it is likely that the *WT1*-enhanced cell viability is due to the markedly reduced expression of *Bax* (Figure 1). As *WT1* is expressed in the GCs of primordial and primary follicles that are poor in FSH receptors, it is unlikely that FSH plays an important role in regulating *Bax* expression at this stage. This, in turn, suggests that *WT1* may change the apoptotic set point by up- or down-regulating the expression of pro-apoptotic proteins, in particular *Bax*. Although the precise mechanism by which other co-expressed factors down-regulate the expression of *Bax* still needs to be determined, the finding that *WT1* suppresses *Bax* accumulation in GCs provides important insight into the regulation of apoptosis during early folliculogenesis.

The anti-apoptotic role of *WT1* during urogenital development was first established by Kreidberg *et al*. [5]. To investigate the role of *WT1* in GC apoptosis, serum was removed to induce spontaneous apoptosis [14]. Consistent with the role of *WT1* in the regulation of ovarian FSH receptor expression in GCs [4], *WT1* (−KTS) seems to play a significant role in enhancing cell survival, although both isoforms increased the proportion of viable cells under serum deprivation conditions (Figure 3A). In addition, the observations of decreased TdT-positive cells, marker of DNA fragmentation (Figure 4), are in agreement with previous reports on the anti-apoptotic action of WT1 in extragonadal cells [5]. Another important regulator of the apoptotic process is the *caspase* family of proteases [18]. Increased permeability of mitochondria under the influence of *Bax* results in the activation of *caspase*s 3 and 7 as the final common pathway of apoptosis [24,30]. In addition to confirming *Bax* regulation by *WT1* in GCs, we demonstrated anti-apoptotic effects of *WT1* (−KTS). According to a previous report, *WT1* (−KTS) induced apoptosis by up-regulating *caspase* 3 in osteosarcoma cells [31]. Conversely, *WT1* (−KTS) was found to inhibit apoptosis in GCs by down-regulating *caspase* 3 as strongly as FSH (Figure 3B). We also observed an anti-apoptotic effect of *WT1* (+KTS), although it was not statistically significant. *WT1* (+KTS) has been reported to inhibit apoptosis by down-regulating *Bax* in several human leukemic cells [11]. However, *WT1* (+KTS) may affect GC survival via a different mechanism from *WT1* (−KTS), as it played no role in the regulation of *Bax* expression (Figure 1).

To our knowledge, this is the first study demonstrating the role of *WT1* in GC apoptosis and survival. Our observations corroborate previous reports on the anti-apoptotic role of *WT1* in non-gonadal cells [11]. We suggest that *WT1* activation is necessary to maintain GC survival during the early stages of follicle development, and that *WT1* can influence apoptosis at a number of points, including regulation of an upstream activator (*Bax*), and downstream effectors (*caspase*s 3 and 7). Thus, *WT1* may be a key regulator responsible for enhancing GC survival, with a fine-tuning role during the early stages, which take place in a relatively poor vascular environment similar to serum starvation. As more blood supply reaches the follicles and factors from the serum perfuse the cells, *WT1* may counteract some pro-apoptotic factors until the cells can sufficiently respond to FSH. A better understanding of how apoptosis is regulated during the early FSH-independent follicular stages may improve our ability to manipulate fertility and the timing of the menopause.

## Conclusion

These findings suggest that activation of *WT1* is necessary for maintenance of GC survival during early stage of follicles and *WT1* can play a role in protecting apoptosis through the regulation of upstream activator (*Bax*), as well as through regulation of downstream effecter (*caspase*s 3 and 7). Collectively, our findings allow us to draw the conclusion that the fate of the follicles during their development may be determined by the changing pattern of *WT1* and FSH receptor expression.

## References

[1] Hirshfield AN (1991). Development of follicles in the mammalian ovary. Int Rev Cytol.

[2] Chun SY, McGee EA, Hsu SY, Minami S, LaPolt PS, Yao HH, Bahr JM, Gougeon A, Schomberg DW, Hsueh AJ (1999). Restricted expression of *WT1* messenger ribonucleic acid in immature ovarian follicles, uniformity in mammalian and avian species and maintenance during reproductive senescence. Biol Reprod.

[3] Hsu SY, Kubo M, Chun SY, Haluska FG, Housman DE, Hsueh AJ (1995). Wilms’ tumor protein *WT1* as an ovarian transcription factor, decreases in expression during follicle development and repression of *inhibin-alpha* gene promoter. Mol Endocrinol.

[4] Yoon O, Roh J (2012). Regulation of FSH receptor expression by the Wilms’ tumor 1 gene product (*WT1*) in immature rat granulosa cells. Mol Reprod Dev.

[5] Kreidberg JA, Sariola H, Loring JM, Maeda M, Pelletier J, Housman D, Jaenisch R (1993). WT-1 is required for early kidney development. Cell.

[6] Rao MK, Pham J, Imam JS, MacLean JA, Murali D, Furuta Y, Sinha-Hikim AP, Wilkinson MF (2006). Tissue-specific RNAi reveals that *WT1* expression in nurse cells controls germ cell survival and spermatogenesis. Genes Dev.

[7] Williams GT, Smith CA (1993). Molecular regulation of apoptosis, genetic controls on cell death. Cell.

[8] Drummond IA, Rupprecht HD, Rohwer-Nutter P, Lopez-Guisa JM, Madden SL, Rauscher FJ, Sukhatme VP (1994). DNA recognition by splicing variants of the Wilms’ tumor suppressor, *WT1*. Mol Cell Biol.

[9] Wang ZY, Qiu QQ, Gurrieri M, Huang J, Deuel TF (1995). *WT1*, the Wilms’ tumor suppressor gene product, represses transcription through an interactive nuclear protein. Oncogene.

[10] Mayo MW, Wang CY, Drouin SS, Madrid LV, Marshall AF, Reed JC, Weissman BE, Baldwin AS (1999). *WT1* modulates apoptosis by transcriptionally upregulating the *bcl-2* proto-oncogene. EMBO J.

[11] Ito K, Oji Y, Tatsumi N, Shimizu S, Kanai Y, Nakazawa T, Asada M, Jomgeow T, Aoyagi S, Nakano Y, Tamaki H, Sakaguchi N, Shirakata T, Nishida S, Kawakami M, Tsuboi A, Oka Y, Tsujimoto Y, Sugiyama H (2006). Antiapoptotic function of 17AA(+)*WT1* (Wilms’ tumor gene) isoforms on the intrinsic apoptosis pathway. Oncogene.

[12] Loeb DM (2006). *WT1* influences apoptosis through transcriptional regulation of *Bcl-2* family members. Cell Cycle.

[13] Mosmann T (1983). Rapid colorimetric assay for cellular growth and survival, application to proliferation and cytotoxicity assays. J Immunol Methods.

[14] Tilly JL, Billig H, Kowalski KI, Hsueh AJ (1992). Epidermal growth factor and basic fibroblast growth factor suppress the spontaneous onset of apoptosis in cultured rat ovarian granulosa cells and follicles by a tyrosine kinase-dependent mechanism. Mol Endocrinol.

[15] Harman SM, Louvet JP, Ross GT (1975). Interaction of estrogen and gonadotrophins on follicular atresia. Endocrinology.

[16] Hirshfield AN (1986). Effect of a low dose of pregnant mare’s serum gonadotropin on follicular recruitment and atresia in cycling rats. Biol Reprod.

[17] Hughes FM, Gorospe WC (1991). Biochemical identification of apoptosis (programmed cell death) in granulosa cells, evidence for a potential mechanism underlying follicular atresia. Endocrinology.

[18] Markström E, Svensson EC, Shao R, Svanberg B, Billig H (2002). Survival factors regulating ovarian apoptosis- dependence on follicle differentiation. Reproduction.

[19] McGee EA, Hsueh AJ (2000). Initial and cyclic recruitment of ovarian follicles. Endocr Rev.

[20] Asselin E, Xiao CW, Wang YF, Tsang BK (2000). Mammalian follicular development and atresia, role of apoptosis. Biol Signals Recept.

[21] Tilly JL, Kowalski KI, Johnson AL, Hsueh AJ (1991). Involvement of apoptosis in ovarian follicular atresia and postovulatory regression. Endocrinology.

[22] Roh J, Bae J, Lee K, Mayo K, Shea L, Woodruff TK (2009). Regulation of Wilms’ tumor gene expression by nerve growth factor and follicle-stimulating hormone in the immature mouse ovary. Fertil Steril.

[23] Antonsson B, Martinou JC (2000). The *Bcl-2* protein family. Exp Cell Res.

[24] Oltvai ZN, Milliman CL, Korsmeyer SJ (1993). *Bcl-2* heterodimerizes in vivo with a conserved homolog, *Bax*, that accelerates programmed cell death. Cell.

[25] Graidista P, Nawakhanitworakula R, Saekooa J, Dechsukhumc C, Fujise K (2010). Anti-apoptotic function of T-KTS+, T-KTS-, *WT1*+/+ and *WT1*+/− isoforms in breast cancer. Asian Biomed.

[26] Rauscher FJ, Morris JF, Tournay OE, Cook DM, Curran T (1990). Binding of the Wilms’ tumor locus zinc finger protein to the EGR-1 consensus sequence. Science.

[27] Wang ZY, Qiu QQ, Enger KT, Deuel TF (1993). A second transcriptionally active DNA-binding site for the Wilms tumor gene product, *WT1*. Proc Natl Acad Sci U S A.

[28] Hewitt SM, Hamada S, McDonnell TJ, Rauscher FJ, Saunders GF (1995). Regulation of the proto-oncogenes *bcl-2* and c-myc by the Wilms’ tumor suppressor gene *WT1*. Cancer Res.

[29] Tilly JL, Tilly KI, Kenton ML, Johnson AL (1995). Expression of members of the *bcl-2* gene family in the immature rat ovary, equine chorionic gonadotropin-mediated inhibition of granulosa cell apoptosis is associated with decreased *Bax* and constitutive *bcl-2* and bcl-xlong messenger ribonucleic acid levels. Endocrinology.

[30] Lakhani SA, Masud A, Kuida K, Porter GA, Booth CJ, Mehal WZ, Inayat I, Flavell RA (2006). *Caspase*s 3 and 7, key mediators of mitochondrial events of apoptosis. Science.

[31] Morrison DJ, English MA, Licht JD (2005). *WT1* induces apoptosis through transcriptional regulation of the proapoptotic *Bcl-2* family member Bak. Cancer Res.

